# Successful Treatment of Double-Orifice Mitral Stenosis with Percutaneous Balloon Mitral Commissurotomy

**DOI:** 10.1155/2012/315175

**Published:** 2012-06-11

**Authors:** Suresh V. Patted, Prabhu C. Halkati, Sameer S. Ambar, Ameet G. Sattur

**Affiliations:** ^1^Department of Cardiology, KLE University, Belgaum 590010, Karnataka, India; ^2^KLES Prabhakar Kore Hospital and Medical Research Center, Nehru Nagar, Belgaum-590010, Karnataka State, India

## Abstract

Double-orifice mitral valve (DOMV) is an uncommon congenital anomaly, being present in 0.05% of the general population. The isolated occurrence of this anomaly is very rare and, to our knowledge, no data are currently available on the incidence of an isolated DOMV. A DOMV is characterized by a mitral valve with a single fibrous annulus with 2 orifices opening into the left ventricle (LV). Subvalvular structures, especially the tensor apparatus, invariably show various degrees of abnormality. It can substantially obstruct mitral valve inflow or cause mitral valve incompetence. We present a rare case of nineteen-year-old male who underwent percutaneous mitral balloon commissurotomy in stenotic DOMV.

## 1. Introduction

 Double-orifice mitral valve (DOMV) is an uncommon congenital anomaly, being present in 0.05% of the general population [1]. It was first described by Greenfield in 1876 [2]. DOMV is often associated with other congenital heart defects, in particular, atrioventricular septal defects. The isolated occurrence of this anomaly is very rare, and to our knowledge, no data are currently available on the incidence of an isolated DOMV [3]. Atrioventricular septal defect is present in and left heart anomalies including coarctation and ventricular septal defect in 40%. Mitral insufficiency is most commonly present (45% to 50%), followed by normal flow pattern (35%). Severe valvular stenosis may be present in approximately 13% [4]. Trowitzsch et al. classified DOMV into 3 types: complete bridge, incomplete bridge, and hole. The complete bridge type is characterized by the presence of a fibrous tissue visible from the leaflet edge through the valve ring. In the incomplete form, however, the fibrous connection occurs only at the leaflet edge. In the hole type, the secondary orifice with its subvalvular apparatus occurs in the lateral commissure and is visible only at the mid-leaflet level [5]. We recently encountered an incomplete bridge stenotic DOMV. Few case reports are available in literature where Inoue-balloon mitral valvuloplasty (BMV) was successfully used to split the fibrous connection between the leaflets [6]. We present our experience of rare case of percutaneous mitral balloon commissurotomy in stenotic DOMV. 

## 2. Case Report

**Figure 1:**
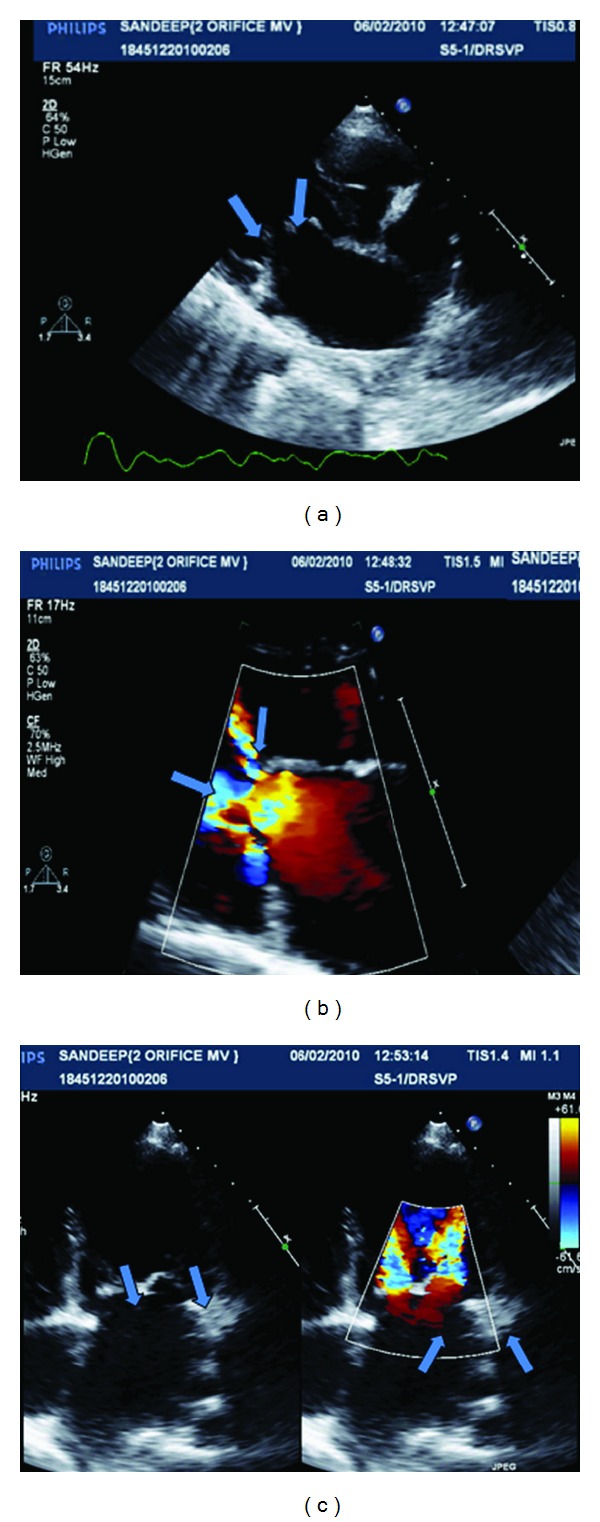


A 19-year-old male patient presented to us with NYHA class two dyspnea. He had previously been diagnosed with rheumatic heart disease and was put on penicillin prophylaxis. His physical examination showed regular pulse and his blood pressure was 120/86 mm Hg cardiac palpation and percussion was unremarkable; auscultation showed an accentuated first heart sound, and diastolic rumbling murmur. Chest X-ray showed evidence of pulmonary venous congestion. Transthoracic and transesophageal 2-dimensional echocardiography (Figure 1) revealed a double-orifice mitral valve of incomplete type at the leaflet level. Both orifice sizes were unequal in our patient, with the anterolateral orifice being much smaller than its counterpart. Surprisingly in our patient there was moderate subvalvular fusion and both commisures were fused contrary to other case reports mentioned in literature. Color doppler examination showed 2 separate mitral diastolic flows with mean gradients of 11 and 13 mm of Hg, respectively. There was no left atrial clot seen by transesophageal echocardiography. 

**Figure 2:**
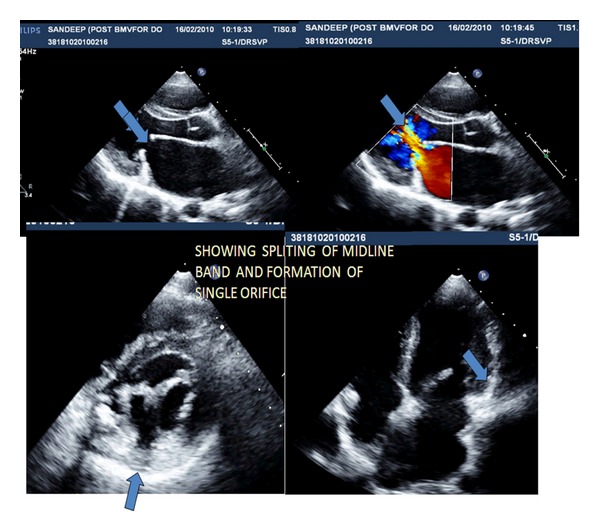


 After informed written consent, BMV was performed using the Inoue-balloon technique. Initially, PA pressure recording was done and LV angiography recorded in RAO view for assessment of mitral regurgitation. Transseptal catheterization and left atrial placement of the balloon catheter were performed in the usual manner [7]. As balloon crossing of the anterolateral orifice proved difficult in the previous cases mentioned in literature, we crossed balloon through posteromedial orifice which was readily accomplished [6]. The posteromedial orifices were dilated using the stepwise dilation technique. Balloon catheter selection was based on the balloon reference size derived from the usual height-based formula and 4 mm less size was taken for first dilation. Following each dilation procedure and after confirming no significant mitral regurgitation or leaflet tears by echocardiography, the balloon size was increased by one mm. The procedure was terminated when the waist of the inflated balloon suddenly disappeared, and echocardiography confirmed separation of the mitral valve septation, resulting in a single enlarged orifice (Figure 2). At the end of the procedure, the mean left atrial pressure, transmitral pressure gradient, and mean pulmonary artery pressure decreased as shown in Table 1. Repeat left ventriculography showed no increase in mitral regurgitation. Patient remained asymptomatic at the latest follow-up visits at one month after BMV. Follow-up echocardiography showed no mitral valve restenosis as defined by 50% reduction in the mitral valve area.

## 3. Discussion

 A DOMV was first described by Greenfield in 1876. Banerjee et al. reported the incidence of DOMVs as 0.05% in a total of 13,400 new patients undergoing echocardiographic evaluation. An isolated DOMV is very rare, and to our knowledge, no data are currently available on the incidence of an isolated DOMV [8]. A DOMV is characterized by a mitral valve with a single fibrous annulus with 2 orifices opening into the left ventricle (LV). Subvalvular structures, especially the tensor apparatus, invariably show various degrees of abnormality. Although double orifice mitral valve may allow normal hemodynamic flow between the left atrium and LV, it can substantially obstruct mitral valve inflow or cause mitral valve incompetence. There is no case report available in literature about rheumatic involvement of DOMV. Most of the case series have observed that there was no commissural fusion in DOMV; as well as very less subvalvular apparatus fusion [6]. But in our case, there was significant subvalvular fusion and both commissures were fused. Taken into consideration high prevalence of RHD in India; this can be first case report mentioning about rheumatic involvement in DOMV. But at this time, it is very difficult to prove chronic RHD from domv.

 We have demonstrated that Inoue BMV can be safely and successfully applied to split the fibrous septation in DOMV, resulting in a single enlarged mitral orifice. Our case demonstrates some important tips regarding BMV in such case. First, balloon size used is 4 mm less than normal reference diameter derived from height-based formula. This will prevent tear of leaflets and excessive development of mitral regurgitation. Second; crossing of posteriomedial orifice is easier as it is more caudally situated. Crossing of anteriolateral orifice is not as difficult as previous reports mentioned. All three times we were able to cross anterolatteral orifice without much difficulty and dilatation of which resulted in breaking of midline fibrous band which leads to successful BMV and single enlarged orifice.

 In summary, in symptomatic patients with stenotic DOMV of incomplete bridge type, Inoue BMV with stepwise dilations applied only to the posteromedial orifice appears to be a safe and effective therapeutic modality.

## Figures and Tables

**Table 1:** 

Parameters	PRE-BMV	POST-BMV
LA pressure	(20)	(5)
LV pressure	100/5	110/4
MV gradient (mean)	15 mm Hg	1 mm Hg
MR	Grade 1 MR	Grade 1 MR
Aorta	101/57 (78)	110/67 (80)
